# High femoral anteversion in osteoarthritic knees, particularly for severe valgus deformity

**DOI:** 10.1186/s10195-022-00653-8

**Published:** 2022-08-16

**Authors:** Changzhao Li, Yongheng Ye, Suiwen He, Dongliang Xu, Peiheng He

**Affiliations:** Department of Joint Surgery, Guangdong Provincial Key Laboratory of Orthopedics and Traumatology, The First Affiliated Hospital of Sun Yat-sen University, Guangzhou, 510080 Guangdong Province China

**Keywords:** Femoral anteversion, Femoral trochlea, Lower-extremity deformities, Osteoarthritic, Total knee arthroplasty

## Abstract

**Objective:**

Increased femoral anteversion (FA) has been correlated with less varus deformities in osteoarthritic (OA) knees, but the relationship between FA and the degree of valgus deformity in osteoarthritic (OA) knees is still largely unknown. We aimed to thoroughly analyze the distribution of FA in relation to varus or valgus deformities of the lower extremity in OA knees, and to further clarify the relationship between FA and trochlear morphology.

**Methods:**

235 lower extremities with OA knees were divided into five groups according to the mechanical tibiofemoral angle: excessive valgus (< − 10°), moderate valgus (− 10° to − 3°), neutral (− 3° to 3°), moderate varus (3° to 10°), and excessive varus (> 10°). FA (measured using the posterior condylar axis [pFA] and the transepicondylar axis [tFA]) was measured, and the relationships of FA to the mechanical tibiofemoral angle and femoral trochlear morphology were identified.

**Results:**

Excessive FA (pFA ≥ 20°) was observed in 30.2% of all patients and in 58.8% of patients in the excessive valgus group. pFA showed a strong correlation with mechanical tibiofemoral angle (*p* = 0.018). Both the pFA and the tFA of patients in the excessive valgus group were greater than those in other four groups (all *p* ≤ 0.037). There were significant correlations between tFA and trochlear parameters, including the sulcus angle (SA), lateral trochlear slope (LTS), and medial trochlear slope (MTS) (all *p* ≤  0.028).

**Conclusion:**

High FA is prevalent, particularly in severe valgus knees, and FA is significantly related to the femoral trochlear morphology in OA knees. With the aim of improving the patellofemoral prognosis and complications, high FA should be considered during total knee arthroplasty.

## Introduction

Patellofemoral complications (anterior knee pain, patellar maltracking) are common, with an incidence ranging from 5 to 12%, after total knee arthroplasty (TKA) for osteoarthritic (OA) knees [11]. These complications occur owing to excessive or uneven patellofemoral contact pressure or abnormal patellofemoral kinematics after TKA [13]. Accurate rotational alignments of the femoral component are fundamental to achieving satisfactory tibiofemoral and patellofemoral kinematics [4]. However, most studies on TKA have focused on the rotational profile of the distal femur alone. Only a few clinical studies have investigated the relationship of local rotational characteristics to the overall lower-extremity alignment, particularly the relative rotational positions of the femoral neck and distal femur in OA knees, namely femoral anteversion (FA).

Increased FA, which is defined as increased internal rotation of the distal femoral condyles relative to the femoral neck in the transverse plane, has been considered a predisposing factor for lateral patellar dislocation and one of the major risk factors for disparities between the patella and the trochlear groove [2], which affects patellar tracking and the patellofemoral joint contact pressure [5]. Previous studies have found that FA correlates to the coronal deformities of OA knees, but their relationships are still controversial [2, 4, 19]. Therefore, a thorough evaluation of the influence of FA variations in OA knees may help achieve satisfactory patellofemoral outcomes after TKA. Also, how FA influences the patellofemoral joint in OA knees is largely unknown.

Given the insufficient data in the literature, we aimed to analyze the distribution of FA according to coronal alignment and to further investigate how FA affects the parameters of femoral trochlear morphology in OA knees. We hypothesized that (i) a higher prevalence of abnormal FA (≥ 20°) can be observed in valgus than in varus deformities and (ii) FA would also show significant correlations with parameters related to the femoral trochlea in OA knees.

## Materials and methods

### Study population

After obtaining institutional review board approval, we performed this retrospective study in patients who underwent primary TKA for knee osteoarthritis (Kellgren–Lawrence grades 3–4) between April 2015 and December 2020. Patients were included only when they had both preoperative standing full-limb anteroposterior radiography and axial computed tomography (CT) images of the femur and tibia. Patients were excluded if they met the following criteria: patellofemoral instability or dislocation, posttraumatic osteoarthritis, previous fracture or surgery of the lower extremity, neuromuscular dysfunction, rheumatic arthritis, or other types of inflammatory arthritis. Finally, a total of 145 patients (20 males and 125 females) providing 235 lower extremities were included in this study. The mean age and body mass index of the included patients were 67.8 years (47–87 years) and 26.4 kg/m^2^ (17.5–34.1 kg/m^2^), respectively.

### Radiographic assessments

**Fig. 1:**
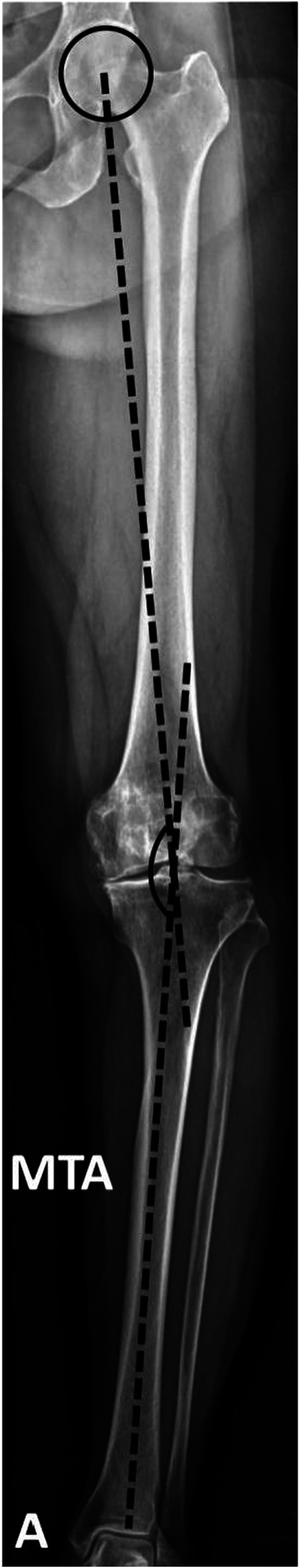
Measurements of the lower extremity deformity (MTA, the angle between the mechanical femoral and tibial axes)

Coronal alignment of the lower extremity was measured using bilateral standing radiography (Philips Medical Systems, Best, Netherlands) of the entire lower extremity (Fig. 1) CT (SOMATOM Force; Siemens Inc., Munich, Germany) examinations were performed with the lower extremities fixed in neutral rotation in a supine position with the knees extended [4]. A specially developed CT protocol scanning including the femur and tibia was used [21, 22, 25]. The slice thickness of the axial CT images was 1 mm.

**Fig. 2:**
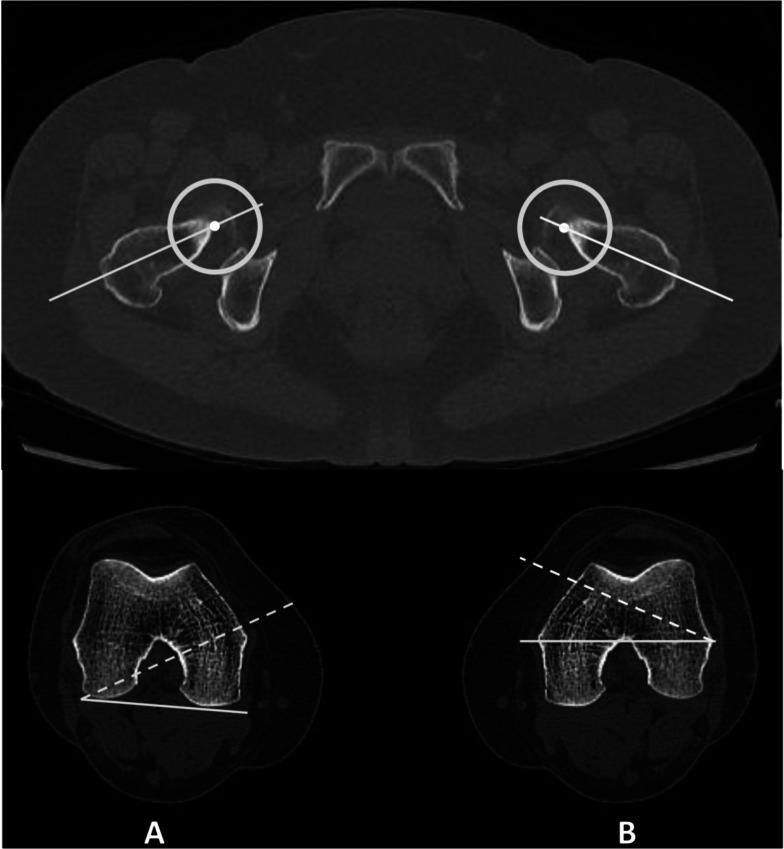
Measurements of the FA using two methods: **A** pFA (angle formed between the lines intersecting the femoral neck axis and the PCL); **B** tFA (angle formed between the lines intersecting the femoral neck axis and the TEA). Positive values indicate femoral anteversion

**Fig. 3:**
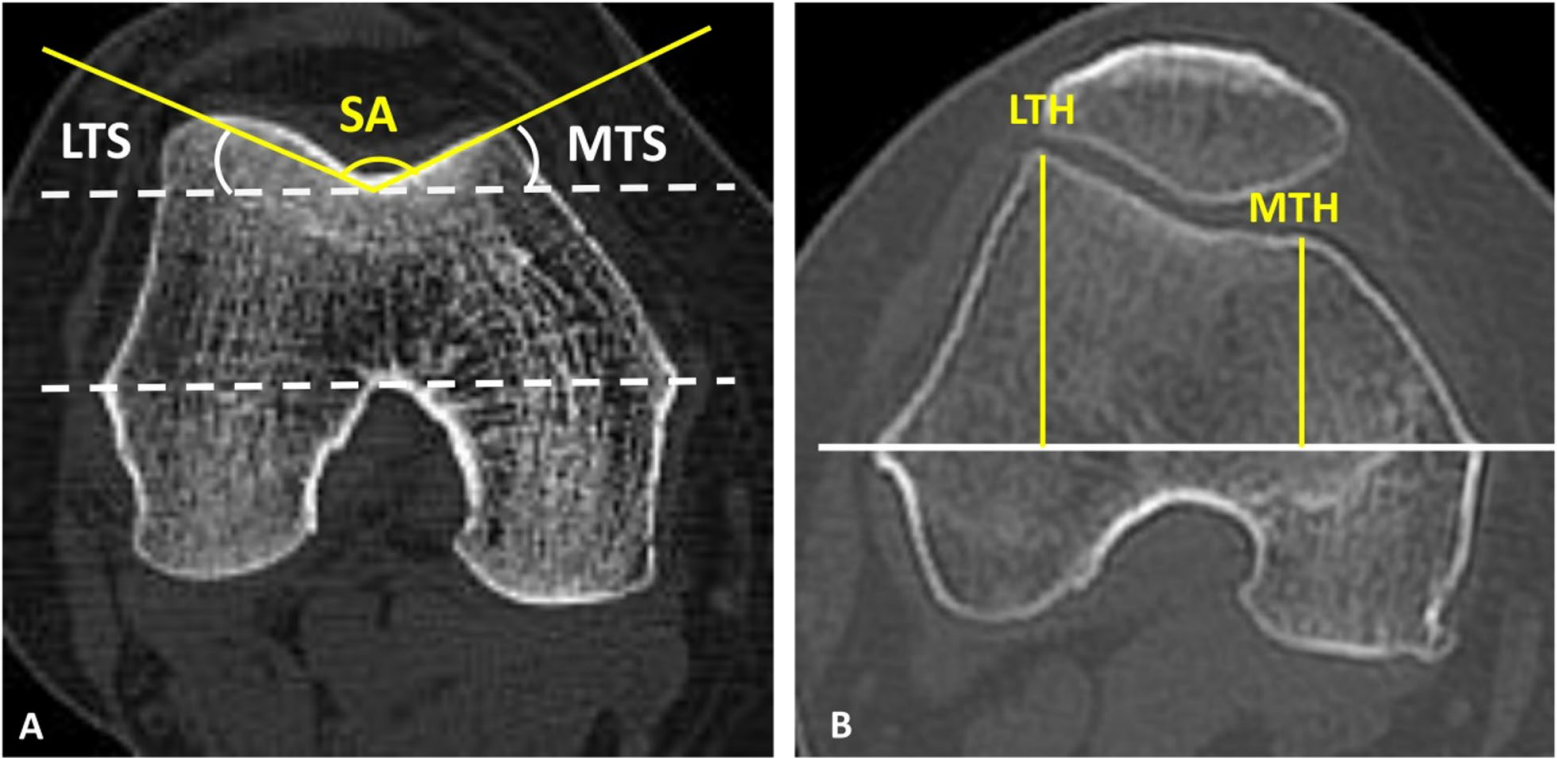
Measurements of the morphology of the femoral trochlea. **A** SA: angle formed by the deepest point of the trochlear groove and the top points of the lateral and medial anterior condyles. LTS and MTS: angle formed between the TEA and the line connecting the deepest point of the trochlear groove and the top point of the lateral or medial anterior condyle, respectively. **B** LTH and MTH: length of the perpendicular line connecting the TEA and the top point of lateral or medial anterior condyle, respectively.

FA was separately measured between the femoral neck axis and the posterior condylar axis (pFA) and between the femoral neck axis and the clinical transepicondylar axis (tFA) [4] (Fig. 2). As for the morphological parameters of the femoral trochlea, the sulcus angle (SA) was the angle formed by the deepest point of the trochlear groove and the top points of lateral and medial anterior condyles [9]. The medial and lateral trochlear slopes (MTS, LTS) were those between the TEA (Transepicondylar axis) and the line connecting the deepest point of the trochlear groove and the top points of the lateral or medial anterior condyle, respectively[9]. The medial and lateral trochlear heights (MTH, LTH) were the lengths of the perpendicular lines connecting the TEA with the top points of the lateral and medial anterior condyles, respectively [18]. The ratio of the LTH and the MTH was defined as the bianterior ratio (BAC) [18]. To minimize the impact of bone size, the MTH and LTH values relative to body height were calculated (MTH-BH and LTH-BH, respectively) (Fig. 3). All parameters were measured using a picture archiving and communication system, with minimum detectable changes of 0.1° in angle measurement and 0.1 mm in length measurement [3]. All measurements were performed by two independent observers (two senior orthopedic residents). The observers repeated the measurements two times with a 3-week interval to minimize learning effects after the initial measurements. To assess the inter- and intraobserver reliability of the measurements, the interclass correlation coefficients of all parameters in all patients were used. All intraclass correlation coefficients of the interobserver and intraobserver reliabilities were high (Table 1).

**Table 1:** Intraclass correlation coefficients of the interobserver and intraobserver error in all parameters

	Intraobserver		Interobserver	
	ICC	95% CI	ICC	95% CI
Femoral anteversion*				
pFA	0.971	0.945 to 0.982	0.933	0.893 to 0.956
tFA	0.983	0.978 to 0.987	0.943	0.875 to 0.960
Mechanical alignment of lower extremity*				
MTA	0.996	0.994 to 0.997	0.917	0.866 to 0.946
Morphology of the femoral trochlea*				
SA	0.925	0.835 to 0.959	0.819	0.762 to 0.885
LTS	0.850	0.640 to 0.921	0.803	0.714 to 0.853
MTS	0.896	0.764 to 0.944	0.795	0.727 to 0.845
LTH	0.908	0.853 to 0.927	0.902	0.796 to 0.940
MTH	0.899	0.667 to 0.958	0.875	0.639 to 0.932
LTH-BH	0.892	0.837 to 0.918	0.874	0.779 to 0.921
MTH-BH	0.878	0.621 to 0.948	0.873	0.687 to 0.918
BAC	0.790	0.698 to 0.838	0.776	0.628 to 0.806

*ICC* intraclass correlation coefficient, *pFA* femoral anteversion based on the posterior condylar line, *tFA* femoral anteversion based on the clinical transepicondylar axis, *MTA* mechanical tibiofemoral angle, *SA* sulcus angle, *LTS* lateral trochlear slope, *MTS* medial trochlear slope, *LTH* lateral trochlear height, *MTH* medial trochlear height, *LTH-BH* lateral trochlear height relative to body height, *MTH-BH* medial trochlear height relative to body height, *BAC* bianterior condyle ratio

*The values given are the intraclass correlation coefficient and the 95% confidence interval

### Statistical analysis

All statistical analyses were performed using SPSS (version 24.0; SPSS Inc., Chicago, IL, USA). All quantitative data in this study passed the test of normality (Kolmogorov–Smirnov test). Pearson correlation analysis was used to determine the relationships of FA to the mechanical alignment of the lower extremity and the femoral trochlear morphology. One-way analysis of variance and post hoc analysis were performed to determine the statistical significance of the differences in the degree of FA among five coronal alignment groups: excessive valgus (< − 10°), moderate valgus (− 10° to − 3°), neutral (− 3° to 3°), moderate varus (3° to 10°), and excessive varus (> 10°). A stepwise regression model using tFA and MTA was constructed. Sample size was calculated for a desired power of 80% and an *α* value of 0.05, and an estimated sample size of 216 was deemed to be required [19]. A *p* value of < 0.05 was considered statistically significant.

## Results

**Fig. 4:**
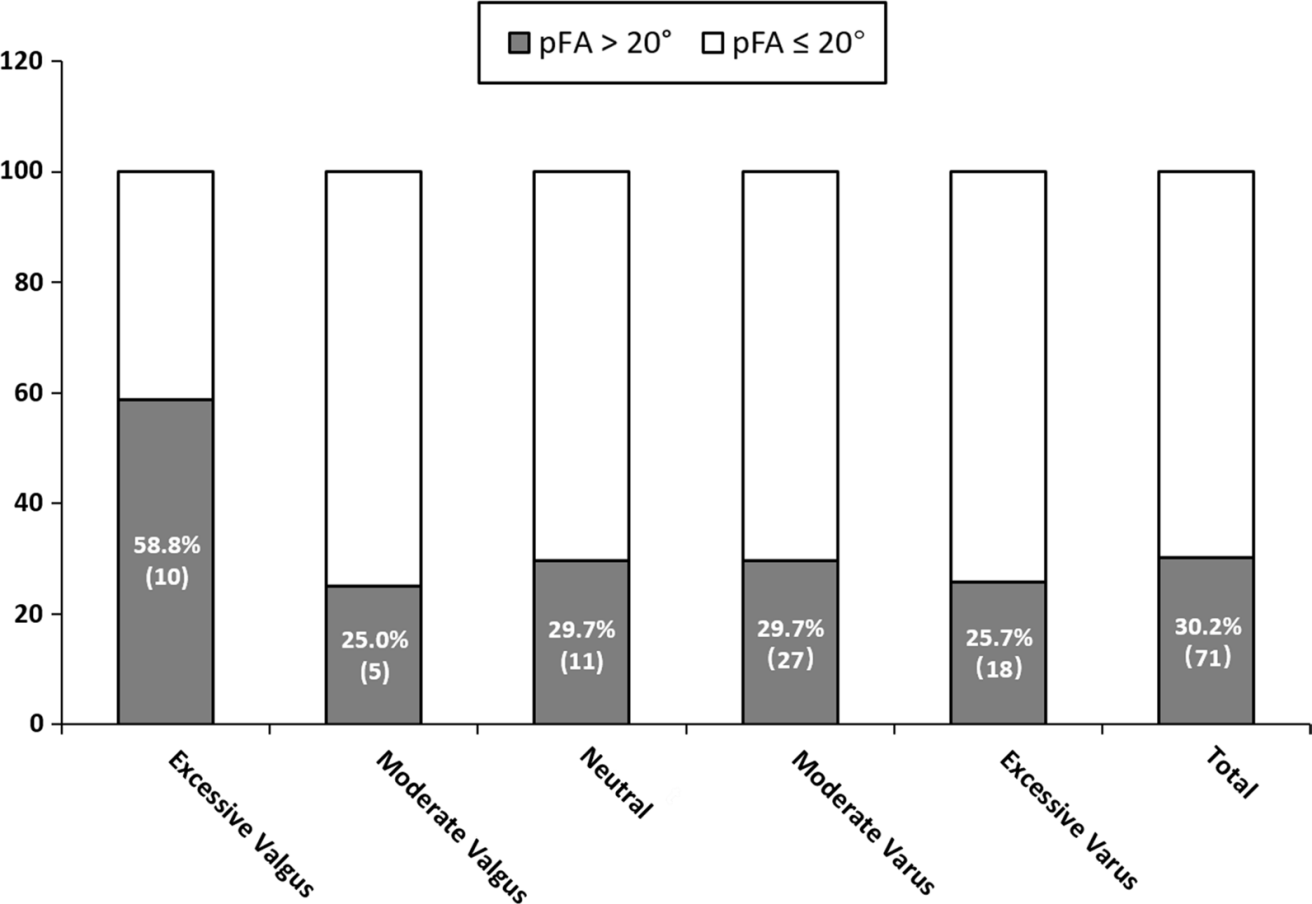
Numbers and ratios of the patients with pFA ≥ 20° and pFA < 20°

**Fig. 5:**
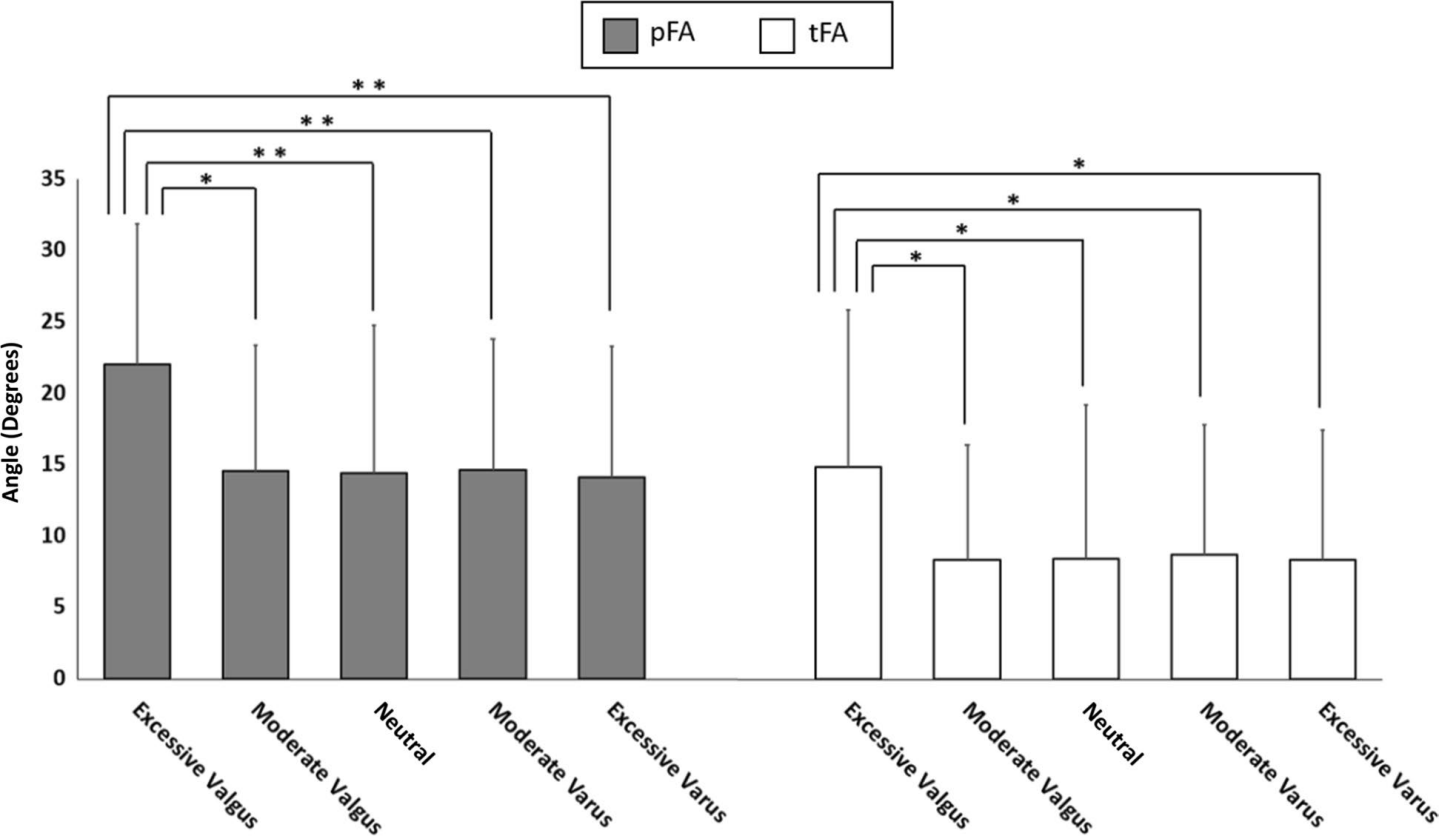
Comparison of radiographic parameters between the groups divided by mechanical tibiofemoral axis. Both pFA and tFA were increased in the excessive valgus group compared to all other groups. *p < 0.05; **p < 0.01

An abnormal FA (≥ 20°) was observed in 30.2% of all patients and in 58.8% of patients in the excessive valgus group (Fig. 4). pFA negatively correlated with coronal alignment (*r* = − 0.154, *p* = 0.018; Table 2). In the subgroup analysis, pFA was, on average, approximately 7.41° to 7.95° greater in the excessive valgus group than in the other four groups (all *p* ≤ 0.016) (Fig. 5). Meanwhile, although no statistically significant correlation was found between MTA and tFA (*r* = − 0.118, *p* = 0.071) (Table 2), tFA was, on average, 6.16° to 6.54° greater in the excessive valgus group than in the other four groups (*p* ≤ 0.037) (Fig. 5).

**Table 2:** Pearson correlation coefficients between the mechanical tibiofemoral angle and the femoral anteversion or femoral bowing angle

Parameters	MTA	
	*r* value	*p* value
pFA	− 0.154	0.018*
tFA	− 0.118	0.071

*pFA* femoral anteversion based on the posterior condylar line, *tFA* femoral anteversion based on the clinical transepicondylar axis, *MTA* mechanical tibiofemoral angle

*Assumed to be statistically significant (*p* < 0.05), based on Pearson analysis

**Fig. 6:**
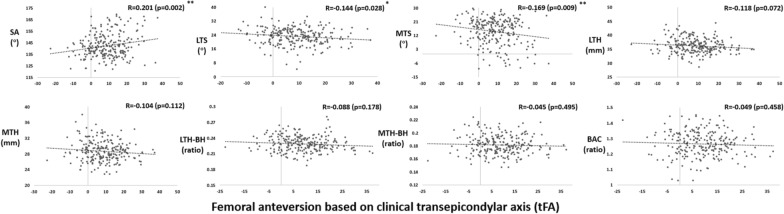
Correlations of tFA with the morphology of the femoral trochlea, including the sulcus angle (SA), lateral trochlea slope (LTS), medial trochlea height (MTH), medial trochlea height (LTH), medial trochlea height (MTH), lateral trochlea height relative to body height (LTH-BH), medial trochlea height relative to body height (MTH-BH), and bianterior condyle ratio (BAC). *p < 0.05; **p < 0.01

With respect to the femoral trochlear morphology, tFA positively correlated with SA (*r* = 0.201, *p* = 0.002) and negatively correlated with LTS (*r* = − 0.144, *p* = 0.028) and MTS (*r* = − 0.169, *p* < 0.009) in OA knees (Fig. 6). Based on the regression analysis for all participants, tFA partly explained the variation of SA (*β* = 0.201, *p* = 0.002), LTS (*β* = − 0.144, *p* = 0.028), and MTS (*β* = − 0.150, *p* = 0.002), while MTA partly explained the variation of MTS (*β* = 0.162, *p* = 0.012) (Table 3). OA knees with pFA ≥ 20° had significantly greater SA (*p* = 0.024) and lower MTS (*p* = 0.032) than OA knees with pFA < 20° (Table 4).

**Table 3:** Stepwise regression analysis and Pearson correlation analysis of the anterior femoral trochlea morphology for OA patients

Morphology of femoral anterior trochlea	Parameter	Regression analysis		Correlation analysis	
		Standardized* β*	*p* value*	*r*	*p* value^#^
Sulcus angle	tFA	0.201	0.002	0.201	0.002
Lateral trochlea slope	tFA	− 0.144	0.028	− 0.144	0.028
Medial trochlea slope	tFA	− 0.150	0.02	− 0.169	0.009
	MTA	0.162	0.012	0.180	0.006

*MTA* mechanical tibiofemoral angle, *tFA* femoral anteversion based on the clinical transepicondylar axis

*The parameter is assumed to contribute statistically significantly to the variation of the anterior femoral trochlea morphology (*p* < 0.05), based on stepwise regression analysis

^#^The parameter is assumed to correlate statistically significantly to the variation of the anterior femoral trochlea morphology (*p* < 0.05), based on Pearson correlation analysis

**Table 4:** *T*-test analysis of morphology of the femoral trochlea with pFA ≥ 20° and pFA < 20°

Parameter	Total ° (SD; range)^a^	pFA ≥ 20° (SD; range)^a^	pFA < 20° (SD; range)^a^	*p*
Sulcus angle	142.3 (10.4; 120.5 to 169.4)	144.6 (12.7; 121.9 to 167.7)	141.3 (9.35; 120.5 to 169.4)	0.024*
Lateral trochlea slope	23.1 (4.89; 4.30 to 39.9)	22.6 (4.39; 12.6 to 32.0)	23.4 (5.08; 4.30 to 39.9)	0.298
Medial trochlea slope	14.6 (8.60; − 9.90 to 29.6)	12.7 (10.0; − 9.20 to 27.6)	15.3 (7.81; − 9.90 to 29.6)	0.032*
Lateral trochlea height	36.1 (2.61; 28.5 to 44.3)	35.8 (1.97; 32.1 to 41.3)	36.3 (2.84; 28.5 to 44.3)	0.210
Medial trochlea height	28.6 (2.56; 22.8 to 38.0)	28.4 (1.93; 23.4 to 34.3)	28.8 (2.79; 22.8 to 38.0)	0.298
Lateral trochlea height relative to body height	0.23 (0.01; 0.19 to 0.28)	0.23 (0.01; 0.21 to 0.28)	0.23 (0.02; 0.19 to 0.28)	0.739
Medial trochlea height relative to body height	0.18 (0.01; 0.15 to 0.22)	0.18 (0.01; 0.15 to 0.22)	0.18 (0.01; 0.15 to 0.22)	0.998
Bianterior condyle ratio	1.27 (0.08; 1.03 to 1.45)	1.26 (0.07; 1.12 to 1.44)	1.27 (0.09; 1.03 to 1.45)	0.607

*pFA* femoral anteversion based on the posterior condylar line, *tFA* femoral anteversion based on the clinical transepicondylar axis

* p < 0.05; ** p < 0.01

^a^Values are given as the median, with the standard deviation and range in parentheses

## Discussion

The main finding of this report is that high FA is prevalent, especially in OA knees with severe valgus deformity. On the other hand, increased FA is associated with flattening of the femoral trochlea.

It is well known that rotational deformities of the distal femur in varus or valgus OA knees should be considered for determining the femoral component rotation when performing TKA [4]. However, only limited data are available about the relationship of rotational deformities of the distal femur to the overall lower-extremity rotational alignment [4, 20]; specifically, quantitative data on the influence of FA during TKA, which are important for interpreting the rotational ability of the distal femur relative to the hip. There is no consensus on the relationship between the mechanical alignment of the lower extremity and the degree of FA in patients with OA knees in the few studies that address this issue [4, 19]. Moon et al. [4] assessed the degree of FA using the transepicondylar axis (tFA) instead of the conventional method of using the posterior condylar axis to eliminate the influence of the posterior condyle [1, 9, 17]. They found no correlation between the coronal alignment and the degree of FA, so they believe that the correlation between mechanical alignment and FA can be mainly attributed to the relationship between the posterior condylar geometry and the coronal alignment. However, Yoon et al. [19] reported that the tFA angle decreased as the degree of varus deformity increased. It should be noted that Moon et al. only generally divided coronal alignment deformities into varus and valgus groups, whereas Yoon et al. further divided the varus group into three subgroups, and almost one-third of the included OA patients had knees with excessive varus deformity (> 10°) in their study. Therefore, it is reasonable to speculate that the change in FA with mechanical alignment may be more evident in knees with excessive varus or valgus deformities. Accordingly, in the current study, we further divided the coronal deformities into five groups, including an excessive valgus group, which was not reported in the aforementioned studies. Our results verified our speculation that tFA was significantly greater in the excessive valgus group than in the other groups. In addition, over half (58.8%) of the OA patients in the excessive valgus group had excessive FA (≥ 20°) [6, 7], and this ratio was significantly greater than for other groups (around one-fourth to one-third). These findings demonstrate that high FA is prevalent, particularly in severe valgus knees. Despite substantial improvements in surgical technologies, the reported incidence of patellofemoral complications still varies from 5 to 12% [11, 23]. Increased FA has been considered a predisposing factor for patellar maltracking, which is one of the major reasons for patellofemoral complications after TKA [2, 5, 13]. Therefore, the prevalence of high FA in OA knees found in the current study may contribute to the high incidence of patellofemoral complications after TKA.

However, only limited knowledge is currently available on the clinical implications of variations in FA for TKA. Mobile-bearing TKA may have potential clinical applications because it can theoretically adjust for rotational malalignment through its self-aligning feature and improve patellar tracking. It has also actually been used for complex patellar maltracking in patients with valgus knee osteoarthritis with permanent patellar dislocation [8]. Additionally, as femoral derotational osteotomy for the treatment of patellar dislocation has been shown to achieve good clinical results [14], it is feasible to implement correction osteotomy at the distal femur for OA knees, as required during TKA operation.

Restoration of the femoral trochlear anatomy influences the functional outcomes of OA knees after TKA [15]. Besides coronal deformity of the lower extremity [24], FA has also been reported to relate to the morphology of the femoral trochlea in previous studies [5, 9, 12]. Liebensteiner et al. [9] investigated the trochlear sulcus and LTS, and their results showed that healthy knees have a shallower trochlear sulcus and a flatter LTS with a higher FA. Reikerhs et al. [12] and Diederichs et al. [5] found a weak or no correlation between FA and the trochlear sulcus or slope in a limited patient group with increased femoral anteversion. Most of these studies focused on healthy knees or knees with femoral trochlear dysplasia, but there has been little study of OA knees in this context. Our results showed that, apart from coronal deformities, increased FA is another important contributor to the flattening of the trochlea, and femoral trochleas with high FA (≥ 20°) were significantly flatter than those with normal FA. This flattening of the trochlea in OA knees with increased FA may be an adaption to the resulting increase in lateral patellofemoral stress. However, during TKA, when a surgeon performs a standard resection of the anterior cortex and uses a prosthesis with a uniform anterior femoral thickness regardless of the variable morphology of the femoral trochlea induced by FA, patellofemoral overstuffing or understuffing may occur after TKA [10, 16]. The present results may provide some useful information for the patellofemoral morphology design of femoral prostheses to accurately replicate femoral trochlea discrepancies according to FA for TKA.

This study had some limitations. First, we included only Asian patients. Racial disparities in FA or in morphological parameters related to the patellofemoral joint may exist, and caution should be used when applying the current results to populations of different ethnicities. Second, the current research lacked clinical results and did not analyze patellofemoral kinematics. Clinical and kinematic analyses of how FA affects the patellofemoral joint after TKA for OA knees should be further investigated to verify the clinical meaning of the current results.

## Conclusion

In OA knees, high FA is prevalent, especially in knees with severe valgus deformity. Increased FA is associated with variable morphology of the femoral trochlea. With the aim of reducing the incidence of patellofemoral complications, the FA should be taken into account during TKA.

## Data Availability

Not applicable.

## Abbreviations

FA: Femoral anteversion
tFA: FA using the transepicondylar axis
pFA: FA using the posterior condylar axis
SA: Sulcus angle
LTS: Lateral trochlear slope
MTS: Medial trochlear slope
MTH: Medial trochlear height
LTH: Lateral trochlear height
OA: Osteoarthritic
TKA: Total knee arthroplasty
CT: Computed tomography
